# Yak milk and its health benefits: a comprehensive review

**DOI:** 10.3389/fvets.2023.1213039

**Published:** 2023-06-28

**Authors:** Qudratullah Kalwar, Xiaoming Ma, Bin Xi, Rashid Ali Korejo, Deepesh Kumar Bhuptani, Min Chu, Ping Yan

**Affiliations:** ^1^Key Laboratory of Animal Genetics and Breeding on Tibetan Plateau, Ministry of Agriculture and Rural Affairs, Lanzhou, Gansu, China; ^2^Shaheed Benazir Bhutto University of Veterinary and Animal Sciences, Sakrand, Sindh, Pakistan; ^3^Key Laboratory of Yak Breeding Engineering, Lanzhou Institute of Husbandry and Pharmaceutical Sciences, Chinese Academy of Agricultural Science, Lanzhou, Gansu, China

**Keywords:** yak, milk, health benefits, therapeutic benefits, humans

## Abstract

Yak milk has various potential health benefits due to its high nutritional content and unique composition. It is an excellent source of protein, essential fatty acids, vitamins, and minerals, which can promote overall health and wellbeing. Yak milk may have potential therapeutic benefits for hypertension, as it contains peptides that have been shown to have antihypertensive effects. Yak milk has also been shown to possess antioxidant properties, which can help protect against oxidative stress and related health problems. Moreover, its fat contains higher levels of beneficial fatty acids, such as conjugated linoleic acid and omega-3 fatty acids, which have been linked to various health benefits, including reducing inflammation, improving heart health, and supporting brain function. Moreover, further research is needed to fully understand the potential health benefits of yak milk, its unique composition and high nutritional content suggest that it may offer numerous health benefits and could be a valuable addition to a healthy diet.

## 1. Introduction

The yak (*Bos grunniens*) is a long-haired mammal that is native to the Himalayan region of South Central Asia, including parts of Tibet, Nepal, Bhutan, and India. It belongs to the family Bovidae, which also includes domestic cattle, bison, and buffalo. Moreover, Yaks are well adapted to living in high altitudes, with thick fur to protect against the cold and low-oxygen environments. They are herbivores, primarily grazing on grasses, herbs, and lichens. In addition to their meat, yaks are also used for their milk, which is high in fat and protein, and for their wool, which is used to make clothing and other textiles (1). Yaks are an important part of the culture and economy of the Himalayan region, and they are also used for transportation, as pack animals, and in traditional ceremonies and festivals (2–5).

Milk is an important source of all basic nutrients required for mammals including human beings (2, 6). Yak produced, around 150–500 kg milk per lactation and its production depends on the breed, age, parity and body condition of the yak; pasture growth pasture quality, raising areas, milking time, milking methods, and other environmental factors (7). Yak milk and its products are the major ingredients of the daily diet of Tibetan herders, particularly for the weak, ill, elderly and young in the areas where yaks graze on the alpine meadows and mountain pastures. Due to a shortage of fruit and vegetables, and limited food resources, yak milk and milk products (butter and cheese) are a vital source of vitamins and major sources of nutrition for Tibetan Herders (7). Yak milk is called natural concentrated milk because of its high fat, protein and lactose, and minerals content during the main lactating period (8, 9). In addition to that, its milk is richer in polyunsaturated fatty acids, protein, casein and fat (10). Besides, to its nutritional value, yak milk and its products may offer functional benefits such as supporting immune function, reducing inflammation, and improving heart health (11). The most recent review paper indicates that yak milk and its derivatives exhibit a wide range of bioactive properties. These include antioxidant effects, which help combat oxidative stress and prevent cellular damage (12). Additionally, yak milk and its derivatives show potential as anticancer agents, displaying properties that may inhibit the growth and spread of cancer cells (12). Furthermore, they have demonstrated antimicrobial activity, which can contribute to the prevention and treatment of microbial infections. Yak milk and its derivatives also exhibit blood pressure-lowering effects, potentially aiding in the management of hypertension (13). These potential benefits have led to increased interest in the use of yak milk and its products as functional foods and dietary supplements. Generally, the rich nutritional and functional properties of yak milk and its products make it a valuable food source that deserves further investigation and exploration. Previously limited studies are available regarding yak milk, hence in this review; we have discussed yak milk and its products, properties, and its beneficial impacts, which might be helpful for future studies (Tables 1–6).

**Table 1:** The fatty acid content of yak milk (72, 73).

Fatty acid	Concentration (g/100 g fat) function	
Butyric acid (C4:0)	3.5–4.5	Butyric acid has anti-inflammatory properties and is beneficial for gut health.
Caprylic acid (C8:0)	1.5–2.5	caprylic acid, has antimicrobial properties and can help maintain a healthygut microbiome.
Capric acid (C10:0)	2.0–3.0	It contributes to its antimicrobial properties and supports the immune system.
Palmitic acid (C16:0)	25–35	It is a primary energy source and contributes to the flavor of dairy products.
Stearic acid (C18:0)	8–12	Contributing to the texture of dairy products.
Oleic acid (C18:1)	24–30	Improve heart health and reduced inflammation.
Linoleic acid (C18:2)	2.5–5.5	It plays a crucial role in maintaining skin health, growth, and development.

**Table 2:** Protein content of yak milk (12, 41, 72, 74–77).

Protein	Concentration (g/100 g milk)
Total protein	4.5–5.5
Casein	3.2–4.0
Whey protein	0.7–1.2
αs1-Casein	0.8–1.2
αs2-Casein	0.3–0.7
β-Casein	1.7–2.3
κ-Casein	0.4–0.7
α-Lactalbumin	0.2–0.4
β-Lactoglobulin	0.4–0.7
Serum albumin (mg/100 g)	77–165
Lactoferrin (mg/kg)	200–700
Immunoglobulins (mg/kg)	100–400

**Table 3:** Macroelements and microelements of yak milk with comparison to other animals (41, 45, 74–77).

Macroelements					
Element	Yak milk	Cow milk	Buffalo milk	Goat milk	Sheep milk
Calcium	1,267 mg/L	1,180 mg/L	880 mg/L	1,330 mg/L	1,340 mg/L
Phosphorus	986 mg/L	935 mg/L	730 mg/L	944 mg/L	870 mg/L
Magnesium	91 mg/L	39 mg/L	42 mg/L	49 mg/L	45 mg/L
Potassium	1,149 mg/L	690 mg/L	704 mg/L	1,090 mg/L	920 mg/L
Sodium	224 mg/L	470 mg/L	120 mg/L	441 mg/L	486 mg/L
Microelements					
Iron	0.61 mg/L	0.05 mg/L	0.04 mg/L	0.19 mg/L	0.11 mg/L
Copper	0.14 mg/L	0.12 mg/L	0.10 mg/L	0.20 mg/L	0.18 mg/L
Zinc	4.78 mg/L	4.70 mg/L	4.02 mg/L	4.31 mg/L	4.30 mg/L
Manganese	0.08 mg/L	0.02 mg/L	0.02 mg/L	0.03 mg/L	0.03 mg/L
Selenium	3.14 μg/L	3.05 μg/L	3.43 μg/L	4.57 μg/L	4.17 μg/L

**Table 4:** Average composition per 100 g of milk of yak, camel, mare, cow, and human milk (10, 12, 13).

Component	Yak	Camel	Mare	Cow	Human
Fat, g	6.5	4.5	1.3	4.0	4.0
Protein, g	5.1	3.5	2.1	3.4	1.9
Lactose, g	4.4	4.4	6.4	4.8	6.5
Minerals, g	0.8	0.7	0.4	0.7	0.2
Solids-non-fat, g	10.4	8.6	9.3	9.0	7.3
Total solids, g	16.9	12.8	10.5	13.3	12.1
Cholesterol, mg	22.0	37.0	4.5	14	20
Saturated FA, g	3.9 2.4	0.4	2.4	1.8	
Monounsaturated FA, g	2.2	1.4	0.3	1.1	1.6
Polyunsaturated FA, g	0.4	0.5	0.5	0.1	0.5

**Table 5:** Concentration of vitamins in milk of yak (41, 54, 74–77).

Vitamins	Concentration (mg/L milk)	Function in the body
Vitamin A	0.6–1.2 mg	Necessary for vision, immune function, and cellular communication.
Vitamin D	0.005–0.015	Helps with the absorption of calcium and phosphorus and supports bone
Vitamin E	1–6	Acts as an antioxidant and supports immune function.
Vitamin K	0.002–0.006	Necessary for blood clotting and bone health.
Vitamin B1	0.2–0.6	Helps convert carbohydrates into energy, and plays a role in nerve function.
Vitamin B2	1.2–2	Helps convert food into energy and supports healthy skin and eyes.
Vitamin B3	0.1–0.25	Plays a role in energy production and helps maintain healthy skin and nerves.
Vitamin B5	0.2–0.5	Plays a role in energy metabolism and hormone production.
Vitamin B6	0.3–0.6	Helps with the metabolism of amino acids and supports nervous system function.
Vitamin B9	0.005–0.015	Plays a role in DNA synthesis and supports healthy fetal development.
Vitamin B12	0.2–0.6	Necessary for nerve function and red blood cell production.

**Table 6:** Amino acid content of yak milk (16, 72).

Amino Acid	Concentration (mg/100 g) function	
Histidine	110–140	Important for the growth and maintenance of tissues and the synthesis of hemoglobin.
Isoleucine	230–290	Involved in muscle metabolism, immune function, and energy production.
Leucine	370–460	Regulates protein synthesis, muscle growth, and repair and helps maintain blood sugar levels.
Lysine	290–360	Lysine is vital for protein synthesis, hormone production, and the absorption of calcium.
Methionine	100–130	Methionine is crucial for the synthesis of proteins, the production of cysteine, and maintaining a healthy liver.
Phenylalanine	200–250	Essential for the synthesis of neurotransmitters like dopamine, norepinephrine, and epinephrine, which play a role in mood regulation and cognitive function.
Threonine	240–300	Supports the immune system, synthesis of proteins, and production of collagen and elastin.
Tryptophan	40–60	A precursor to serotonin, which regulates mood, appetite, and sleep, and is also involved in the synthesis of melatonin.
Valine	260–330	Involved in muscle growth, repair, and energy production.

## 2. Chemical properties of yak milk

Yak milk is a valuable food source with unique chemical properties that make it an attractive alternative to cow’s milk. Its lower fat (5.5%) and lactose content (3.7%), and higher protein (5.9%), mineral and immunoglobulin’s content make it a healthier milk option for people, who are lactose intolerant or looking for a nutritious milk alternative. In addition to that the pH of yak milk is slightly acidic, ranging from 6.3 to 6.8 (14–17). Detailed regarding chemical properties are mentioned below:

### 2.1. The fatty acid content of yak milk

Yak milk is a rich source of various fatty acids (FAs), including both saturated and unsaturated FAs. Saturated fatty acids (SFAs) are the predominant type of fatty acid found in yak milk, accounting for about 65–75% of total fatty acids. The most abundant SFAs in yak milk are palmitic acid (C16: 0) and stearic acid (C18:0). Meanwhile, Monounsaturated fatty acids (MUFAs) make up about 20–25% of the total fatty acids in yak milk. The most common MUFA in yak milk is oleic acid (C18:1). Additionally, polyunsaturated fatty acids (PUFAs) account for a relatively small proportion of the total fatty acids in yak milk, typically around 3–6%. The most abundant PUFAs in yak milk are linoleic acid (C18:2) and alpha-linolenic acid (C18:3). Overall, the fatty acid profile of yak milk is considered to be nutritionally beneficial, as it is low in SFAs and high in MUFAs and PUFAs. This composition has been associated with potential health benefits such as reduced risk of cardiovascular disease and improved lipid metabolism (18–22). Recently, there has been increased attention given to the biological roles of specific fatty acids (FA) found in yak milk fat, beyond their nutritional value. Improving the FA profiles of food has become a priority in many studies, particularly those focusing on conjugated linoleic acid (CLA) and its isomers. These FAs have been shown to have potential health benefits such as antidiabetic and anticarcinogenic effects, as well as a positive influence on immune function (23–25). In addition, eicosapentaenoic acid (EPA) and docosahexaenoic acid (DHA) are considered essential for human health, particularly for proper brain, heart, and retina function. Yak milk fat is particularly unique due to the grazing environment of yaks, and may contain some unique FAs not found in milk fat from other mammals (26–28). The fatty acid composition of yak milk can vary depending on various factors such as diet, season, stage of lactation, and genetics (29) noted seasonal fluctuations in the fatty acid (FA) composition of milk. Similar variations in cis-9,trans-11 CLA and vaccenic acid content have been reported in milk fat from pasture-grazed ewes, as documented in studies by Nudda et al. (30) and Ostrovský et al. (31). The parity of an animal is another important factor that can influence the fatty acid (FA) profile of its milk. In a study by Kelsey et al. (32), differences in conjugated linoleic acid (CLA) content were observed between primiparous and multiparous animals. Similarly, in yak milk, the percentage of unsaturated FAs was found to be higher in multiparous animals compared to primiparous ones, as reported by Peng et al. (33).

### 2.2. Yak milk protein

Yak milk is a rich source of proteins, which are essential for growth, development, and repair of tissues in the body. Caseins proteins account for about 80% of the total protein content in yak milk. Caseins are a group of phosphoproteins that form a gel-like structure when coagulated. They are responsible for the milk’s white color and contribute to the texture and nutritional value of dairy products like cheese and yogurt. Caseins are rich in essential amino acids, making them a valuable source of nutrition. In a previous study, it was reported that yak milk contains around four types of casein proteins, including κ-casein, αs1-casein, αs2-casein, and β-casein. Additionally, the presence of vitamin D-binding protein, retinol-binding protein 4, lactotransferrin, and serotransferrin was observed in yak milk. Notably, the cysteine-rich secretory protein 3 (CRISP-3) precursor, which plays a crucial role in cell apoptosis, and vinculin, a protein that supports cell growth, differentiation, and survival, were found to be up-regulated and down-regulated in high-lactation (HL) yak milk, respectively (34). Whey proteins are the remaining 20–30% of the total protein content in yak milk. They are fast-digesting proteins that provide a quick burst of amino acids to the body. Whey proteins founded in yak milk include beta-lactoglobulin, alpha-lactalbumin, and immunoglobulins. Furthermore, the secondary structural characters of yak whey proteins were different from bovine whey proteins. Yak α-LA had an unstable protein structure and lower Td than bovine α-LA. However, yak β-LG had a higher Td than bovine β-LG.During the dry period, the ability of mammary epithelial cells to synthesize lactose, milk fat, casein, lactalbumin, and lactoglobulin decreases, while the concentration of lactoferrin in mammary secretions increases significantly (35–37). Lactoferrin is a multifunctional protein found in yak milk that has antimicrobial, immunomodulatory, and antioxidant properties. It also has potential health benefits such as improving iron absorption and reducing inflammation (38). Immunoglobulins, also known as antibodies, are proteins that are produced by the immune system to recognize and neutralize foreign substances such as bacteria and viruses. Yak milk contains high levels of immunoglobulins, which can provide passive immunity to newborn calves (39).

### 2.3. Macroelements of yak milk

Yak milk is a nutrient-dense food that contains several important macro elements necessary for human health, including protein, fat, carbohydrates, calcium, and phosphorus. Yak milk is a good source of protein, which is essential for building and repairing tissues in the body, and is particularly important for maintaining muscle mass (40). Yak milk is also rich in fat, which is important for energy production, as well as for the absorption of fat-soluble vitamins. Moreover, Yak milk is a good source of calcium (150-200 mg) and phosphorus (100–150 mg) per 100 mL of milk. Calcium is important for bone health and muscle function. Meanwhile, Phosphorus is essential for bone and teeth health, as well as for energy production and cell signaling (41–43).

### 2.4. Microelements of yak milk

Yak milk is a nutrient-dense food that contains several important microelements, such as zinc, iron, copper, manganese, and selenium. Yak milk is a good source of zinc, with around 1.12 ± 1.21 μg/100 g of milk. Zinc is important for immune function, wound healing, and cell growth and division (44). Yak milk is also a good source of iron, with around 0.56 ± 0.16 μg/100 g of milk. Iron is important for the production of hemoglobin, which carries oxygen in the blood (41). Yak milk is a good source of copper, with around 0.16 ± 0.06 μg/100 g of milk. Copper is important for the production of red blood cells, as well as for maintaining healthy connective tissues (44). Yak milk is a good source of manganese, with around 144 ± 29 μg/100 g of milk. Manganese is important for bone health and energy production (41). Yak milk contains trace amounts of selenium, an important antioxidant that helps to protect cells from damage (41).

### 2.5. Vitamin content in yak milk

Yak milk is a rich source of various vitamins such as A, D, E, B12, and riboflavin, all of which are essential for maintaining good health. Yak milk is a good source of vitamin A, with around 0.6–1.2 mg/ L of milk. Vitamin A is important for maintaining healthy vision, skin, and immune function (45). Yak milk is a good source of vitamin D, with around 0.005–0.015 of vitamin D 100 mg/L of milk. Vitamin D is important for maintaining healthy bones and teeth, as well as for supporting immune function (45–48). Yak milk is a good source of vitamin E, with around 1–6 mg of vitamin E per liter of milk. Vitamin E is an antioxidant that helps to protect cells from damage, and it may also help to support immune function (49). Yak milk is a good source of vitamin B12, with around 0.2–0.6 mg/L of milk. Vitamin B12 is important for maintaining healthy nerve cells and red blood cells, as well as for supporting cognitive function (50). Yak milk is a good source of riboflavin, with around 1.2–2 mg of riboflavin per 1,000 mL of milk. Riboflavin (Vitamin B12) is important for energy production and the metabolism of fats, carbohydrates, and proteins (51).

### 2.6. Nitrogen distribution in yak milk

Nitrogen is important for the growth and repair of tissues in the body and is a component of amino acids, which are the building blocks of proteins. In yak milk, nitrogen is distributed among various compounds such as proteins, peptides, free amino acids, and other nitrogenous compounds. Proteins are the largest nitrogen-containing compound in milk and account for about 80% of the total nitrogen content (10). The distribution of nitrogen in yak milk can vary depending on various factors such as the breed of yak, stage of lactation, and feeding practices. Studies have shown that the concentration of total nitrogen in yak milk can range from 0.72 to 0.94%, with proteins accounting for 58 to 84% of the total nitrogen content. The nitrogen distribution in yak milk exhibits seasonal variation, with higher levels of total nitrogen (TN) observed during warmer seasons (52). Typically, as the environmental temperature rises, the total protein content in milk tends to decrease. This peculiar occurrence may be attributed to the limited availability of food resources during the winter season, which could impact the nutritional composition of the milk (53). In terms of specific amino acids, yak milk contains all essential amino acids, including lysine, histidine, phenylalanine, threonine, isoleucine, methionine, tryptophan, leucine, and valine (10). Yak milk also contains high levels of non-essential amino acids such as glutamic acid and aspartic acid. Overall, nitrogen is an important nutrient found in yak milk and plays a vital role in maintaining overall health and well-being. Moreover, another study revealed that the nitrogen content of milk is important for assessing milk quality and nutritional value (47). Yak milk has a total nitrogen content of around 0.75–1.10%. This includes both protein-bound nitrogen and non-protein-bound nitrogen compounds such as urea and ammonia (51). Protein is the main source of nitrogen in milk, and yak milk has a protein content of around 4.4–4.9%. This protein is rich in essential amino acids, which are important for maintaining muscle mass and supporting growth and development (51). The nitrogen in milk can be divided into several fractions, including casein nitrogen, whey protein nitrogen, and non-protein nitrogen compounds. Yak milk has a higher proportion of casein nitrogen compared to whey protein nitrogen, which contributes to its unique flavor and texture (54).

### 2.7. Amino acids in yak milk

Yak milk contains a variety of essential and non-essential amino acids that are important for human nutrition. Essential amino acids found in yak milk include leucine, isoleucine, valine, lysine, methionine, phenylalanine, threonine, and tryptophan. Non-essential amino acids present in yak milk include alanine, arginine, aspartic acid, cysteine, glutamic acid, glycine, histidine, proline, serine, and tyrosine. The amino acid composition of yak milk can vary depending on factors such as diet, stage of lactation, and location. Yak milk is considered a good source of amino acids and has a similar amino acid profile to cow’s milk. Some studies have shown that the protein in yak milk has higher quality than cow’s milk, with a higher content of essential amino acids and better digestibility (52, 53, 55, 56). Meanwhile, it has been reported that the predominant amino acids were glutamate (20%), proline (10%), lysine (10%) and leucine (10%), of which the essential amino acids accounted for 48% of the total amino acids in Pamir yak milk (57).

## 3. Enzymes involved in yak milk

Yak milk contains various enzymes, including proteases, lipases, amylases, and lactases. These enzymes play an essential role in the digestion of milk proteins, fats, carbohydrates, and lactose. Proteases break down proteins, lipases break down fats, amylases break down carbohydrates, and lactases break down lactose. The enzymes in yak milk make it more easily digestible and may provide additional health benefits. Overall, yak milk is a nutrient-rich beverage that contains a variety of enzymes that can be beneficial for human health (58, 59). Furthermore, a comparative study showed that the activity of some hydrolases and oxidoreductases, such as acid phosphatase, alkaline phosphatase, lipase, catalase, and superoxide dismutase, are much higher in yak milk than in milk from Holstein cows. These enzymes could facilitate essential physiological functions by eliminating excessive reactive oxygen species (free radicals), which then do not exceed the levels tolerated in the bodies of high-altitude-dwelling Tibetans (60–64).

## 4. Yak milk products

Yak milk is a valuable source of nutrition for human consumption, but it also has potential as a source of valuable products. Despite living on the highest plateau in the world where high-altitude residence diseases such as premature aging, edema, atherosclerosis, and cancer are common, Tibetans have managed to maintain good health with few reported health issues. Their diet, which mainly consists of simple dairy products derived from yaks such as milk, butter, yoghurt, cheese, and qula (a type of defatted, acidified, and dried milk) (11, 64). Foods and drinks that have undergone fermentation offer a multitude of nutritional and therapeutic benefits. Lactic acid bacteria (LAB) are an important component of fermented milk products and play a significant role in promoting good health. LAB strains such as *L. acidophilus* and *Bifidobacteria spp* are frequently used in probiotic dairy foods for their beneficial effects on gut health. To ensure that cultured products are marketed as healthy, they must meet the recommended standard of containing a minimum of 10^6^ cfu/g at the time of consumption. Here are some examples of milk products that can be derived from yak milk, along with references to relevant studies:

### 4.1. Yak cheese

Yak milk cheese can be made using the same process as cow’s milk cheese, by adding rennet or another coagulating agent to the milk to separate the curds and whey. Yak milk cheese has a distinct flavor and texture, with a nutty, sweet taste and a crumbly texture. Some popular types of yak milk cheese include chhurpi, which is a hard, dried cheese commonly eaten in Nepal and Bhutan, and shosha, a soft cheese used in traditional Tibetan dishes. Yak milk is used to produce a variety of traditional cheeses in regions where yaks are raised, such as Tibetan cheese and Bhutanese cheese. These cheeses are known for their unique flavors and nutritional properties, and have gained popularity outside of their regions of origin (65, 66).

### 4.2. Yak yogurt

Yak milk yogurt can be used as a probiotic due to the presence of beneficial bacteria in the yogurt. Probiotics are live microorganisms that provide health benefits when consumed in adequate amounts, and yogurt is a popular source of probiotics. Yak milk yogurt is made by fermenting yak milk with bacterial cultures, typically including *Lactobacillus delbrueckii* subsp. *bulgaricus and Streptococcus thermophilus* (38, 67–70). These bacteria convert lactose, the main sugar in milk, into lactic acid, which causes the milk to thicken and become yogurt. During the fermentation process, additional beneficial bacteria such as *Lactobacillus acidophilus, Bifidobacterium bifidum, and Lactobacillus casei can* also be present in yak milk yogurt. These bacteria can survive through the digestive system and reach the colon, where they can provide health benefits such as improving gut health, boosting the immune system, and reducing inflammation. Besides, Yak milk yogurt, is higher in fat and protein content compared to cow milk yogurt. Previous findings demonstrated that yak milk yogurt has potential as a functional food, due to its antioxidant and anti-inflammatory properties (68).

### 4.3. Yak milk powder

Yak milk powder is made by evaporating the moisture from fresh milk, which results in a fine, dry powder that can be easily stored and transported. Yak milk can be processed into powder form, which has a longer shelf life and can be used in a variety of food products. It can be used in a variety of ways, such as in cooking and baking, or as a nutritious drink when mixed with water. Yak milk powder is often used by people living in high-altitude regions, where fresh milk may be difficult to obtain or store. It is also becoming more popular in other parts of the world as a specialty food item due to its unique nutritional properties and taste. Studies have shown that yak milk powder has a high nutritional value and is rich in amino acids, minerals, and antioxidants (69).

### 4.4. Yak milk butter

Yak milk butter is made by separating the fat from yak milk and churning it until it becomes butter. Yak milk butter has a slightly different flavor than cow’s milk butter, with a rich, nutty taste. Yak milk butter is often used in traditional Tibetan cuisine, and is also a popular ingredient in certain types of tea. In addition to that, the production process of yak milk butter is similar to that of cow’s milk butter. The cream is separated from the milk and then churned until it solidifies into butter (70). Meanwhile, yak milk butter has higher fat content and lower water content than cow’s milk butter, which makes it more suitable for long-term storage and transportation. Yak milk butter is a good source of fat-soluble vitamins, such as vitamins A, D, E, and K. One study speculated that yak milk butter contains higher levels of vitamin A and vitamin E than cow’s milk butter (38). Another study found that yak milk butter has a higher antioxidant capacity than cow’s milk butter, which may be due to its higher levels of vitamin E (38). Yak milk butter has a distinctive flavor and texture compared to cow’s milk butter; it is more intense buttery flavor and a firmer texture than cow’s milk butter (38).

### 4.5. Kurut

For thousands of years, local herdsmen living in the Qinghai-Tibetan plateau at altitudes above 4,000 m have been manufacturing traditional fermented yak milk called kurut. Kurut is an important food for people of Qinghai (43). Kurut refers to a group of products produced by natural fermentation of yak milk in a specially-treated big jar for 7–8 days at 10–15°C. These conditions are necessary to produce enough acid, alcohol and flavor. A common property of kurut is the presence of alcohol and lactic acid. Kurut contains greater numbers of lactic acid bacteria and yeast than other traditional fermented milks (lactic acid bacteria counts of 9.18 ± 0.851 log cfu/ml; yeast counts of 8.33 ± 0.624 log cfu/ml) (41, 44). This traditional product has higher TS, protein, fat, lactic acid, mineral (e.g., calcium, phosphorus, and magnesium), and vitamin B and C contents than cow milk–based yogurts (44). In addition, traditional fermented yak milk has a high exo polysaccharide content from lactic acid bacteria (LAB), which contributes to its excellent curdling and probiotic effects (71).

## 5. Conclusion

Yak milk has been traditionally consumed by communities living in high-altitude regions, particularly in Central Asia, and it provides essential nutrients and sustenance in challenging environments. Its nutritional composition is distinct, containing higher levels of protein and fat compared to cow’s milk. Yak milk also contains bioactive compounds that have been associated with potential health benefits, including antioxidant and antimicrobial properties. Moreover, Yak milk and its products are a significant part of the daily diet for Tibetan herders, especially for those who are weak, ill, elderly, or young. Besides, to its nutritional value, yak milk and its products may offer functional benefits such as supporting immune function, reducing inflammation, and improving heart health. These potential benefits have led to increased interest in the use of yak milk and its products as functional foods and dietary supplements. This review papers on yak milk not only contribute to our understanding of its nutritional composition and health benefits but also highlight its broader socio-economic and cultural importance. By addressing research gaps and exploring future aspects, we can resolve the full prospective of yak milk, benefiting local communities and the global community as a whole.

## Author contributions

QK and XM: conceptualization and designed the experiments. BX and MC: methodology. QK: wrote the manuscript. MC, RK, and DB: revised the manuscript. PY: supervision and approved the final draft of the manuscript. All authors contributed to the article and approved the submitted version.

## Funding

This study was funded by the National Key R&D Program of China (No. 2022YFD1600102), Project of Gansu Province (20YF8WA031), International Science and Technology Cooperation Base for Grass Feeding Livestock Breeding in Gansu Province, The Agricultural Science and Technology Innovation Program (25-LZIHPS-01), and Foundation for Innovation, Groups of Basic Research in Gansu Province (20JR5RA580).

## Conflict of interest

The authors declare that the research was conducted in the absence of any commercial or financial relationships that could be construed as a potential conflict of interest.

## Publisher’s note

All claims expressed in this article are solely those of the authors and do not necessarily represent those of their affiliated organizations, or those of the publisher, the editors and the reviewers. Any product that may be evaluated in this article, or claim that may be made by its manufacturer, is not guaranteed or endorsed by the publisher.
